# Efficacy of electroacupuncture in assisting postoperative healing of distal radius fractures: study protocol for a randomized controlled trial

**DOI:** 10.1186/s13018-022-03415-8

**Published:** 2022-12-01

**Authors:** Jiani Fu, Xiaowen Cai, Huailiang Ouyang, Chunzhu Gong, Yong Huang

**Affiliations:** 1 School of Traditional Chinese Medicine, Southern Medical University, Guangzhou, China; 2 Department of Traditional Chinese Medicine, Zhujiang Hospital of Southern Medical University, Guangzhou, China; 3 Department of Orthopedics, Shenzhen Pingle Orthopedic Hospital (Shenzhen Pingshan Traditional Chinese Medicine Hospital), Shenzhen, China

**Keywords:** Distal radius fractures, Electroacupuncture, Protocol

## Abstract

**Background:**

Manual reduction and surgical treatment are common methods for distal radius fractures (DRFs). The existing literature suggests that postoperative combined rehabilitation treatment and medication are effective for the healing of DRFs. However, the side effects of these treatments remain to be solved. Previous studies have shown that electroacupuncture (EA) can effectively relieve wrist swelling and improve the joint function in patients with DRFs, but more evidence is needed to prove the effectiveness of EA. This trial aims to explore the efficiency and feasibility of combined EA treatment in postoperative treatment of DRFs compared with routine treatment.

**Methods:**

This is a parallel randomized controlled trial. A total of 222 patients diagnosed with moderate DRFs will be recruited and randomly assigned to an EA group or a routine treatment group at a ratio of 1:1. Routine treatment group will receive medication and rehabilitation. Yangxi (LI 5), Yangchi (TE 4), Yanggu (SI 5), Hegu (LI 4), and Taiyuan (LU 9) will be selected in the EA group for intervention three times a week on the basis of routine treatment. Both groups will receive 8 weeks of treatment and 4 weeks of follow-up. The primary outcome will be ulnar positive variance. The secondary outcomes will include radiographic healing rate, bone strength, hemorheological indices, serum biochemical indicators and inflammatory factors, grip strength, wrist swelling score, patient-rated wrist evaluation, disabilities of arm, shoulder and hand, and visual analogue scale. Outcomes will be evaluated at baseline, postoperative 3rd day, 2nd, 4th, 6th, 8th, and 12th weeks.

**Discussion:**

The results of this study will help establish a more optimized scheme to treat patients with DRFs.

*Trial registration* Chinese Clinical Trial Registry ChiCTR2200062857. Registered on 21 August 2022, www.chictr.org.cn/com/25/showproj.aspx?proj=175567.

## Background

Distal radius fracture (DRF) is one of the most prevalent fractures in the upper extremity, which occurs within 3 cm of the articular surface of the lower radius [1]. DRFs are mainly characterized by local pain, swelling, deformity, and sometimes subcutaneous congestion [2]. In 2014, there were about 580,000 cases of DRFs in China [3]. With the further aging of the population, the disease incidence in China is increasing year by year [4]. This will not only do harm to the health of patients but also affect their life quality [5]. Therefore, it is necessary to study the effective treatment of DRFs.

There are many causes for DRFs, but the most important factor is injury [6]. Manual reduction and surgical treatment are the common methods for the treatment of DRFs [7, 8]. Displaced and intra-articular injuries are usually treated by surgery [9]. Postoperative combined rehabilitation treatment and medication are often required [10, 11]. Although these treatments have a certain effect on the recovery of fractures, there are often problems such as long fracture healing time and strong pain [12, 13]. Therefore, it is expected to explore a combined treatment strategy to solve the above problems, so as to benefit postoperative DRF patients [14].

In recent years, acupuncture has become an adjuvant treatment for dealing with problems following fracture surgery [15]. Researches suggest that acupuncture is helpful for fracture healing and may accelerate the recovery from postoperative complications [16, 17] because acupuncture can promote fracture healing mainly by improving local blood circulation, inhibiting inflammatory factors, and promoting the deposition of minerals and trace elements [18, 19]. A study showed that laser treatment on acupuncture points could relieve pain and improve wrist functionality in patients undergoing rehabilitation therapy after wrist bone fracture [20]. Other studies found that using acupuncture in the postoperative care of patients with hip fractures could reduce pain and inflammation and improve bone strength and bone mineral density [21, 22].

Electroacupuncture (EA) is the application of electrical stimulation to acupuncture needles [23]. EA is good at avoiding some complications or contraindications of traditional acupuncture [24]. One study has proved that EA combined with functional exercise can effectively relieve postoperative pain and improve elbow joint function in patients with comminuted olecranon fractures [25]. Another study has proved that EA can shorten the clinical healing time for the middle and lower third fractures of the tibia and fibula [26]. However, there are few studies on EA promoting the postoperative healing of DRFs. Because of the limited sample size covered in the existing studies, the advantages of EA in promoting fracture healing after DRFs surgery are still uncertain. Therefore, we design a randomized controlled trial to explore the efficiency and feasibility of combined EA treatment in postoperative treatment of DRFs compared with routine treatment.

## Methods

### Objectives

Through this study, we expect to evaluate the efficacy of combined EA treatment in patients with DRFs.

### Study design

**Fig. 1:**
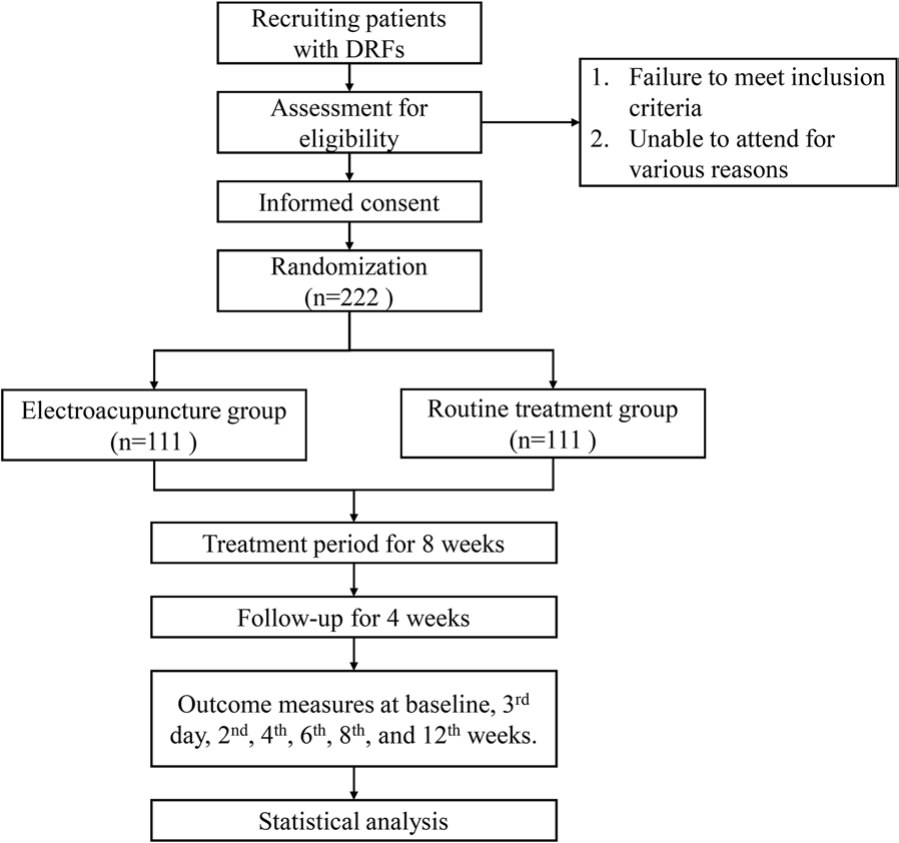
The test procedure

This study is designed as a single-center, randomized, and controlled trial and the main endpoint is the ulnar positive variance at the 8^th^ week after the operation. Eligible participants will be randomly assigned to one of two groups (EA group or routine treatment group) with a ratio of 1:1. The trial procedure is shown in Fig. 1. This protocol is designed according to SPIRIT list [27] and Helsinki Declaration [28].

### Patient recruitment

We will recruit participants for this trial mainly through internet publicity and leaflets in hospitals. The participants interested in this study will be strictly screened according to the inclusion criteria.

### Informed consent

A trained researcher will distribute information sheets about the trial to participants. Participants will know the process and precautions of the whole trial through the information sheets. The researcher will also answer questions from participants about the trial. After the above process is completed, the researcher will provide an informed consent form for every participant who is willing to participate in the trial and invite them to sign the consent form.

### Inclusion criteria

1. Fracture with radius shortening > 3 mm, joint facing dorsal tilt > 10 mm, or significant intra-articular fracture shift or step > 2 mm after manual reduction [29].
2. Surgical treatment with open reduction and internal fixation (ORIF).
3. 18–65 years old.
4. Fracture due to trauma only.
5. First fracture and fresh fracture.
6. Moderate fracture (41 points ≤ Patient-rated Wrist evaluation score < 60 points) [30].
7. Agree to participate and sign the informed consent form.

### Exclusion criteria

1. Open fractures, stable fractures, or old fractures.
2. Severe liver and kidney dysfunction.
3. Patient with thrombocytopenia, bleeding tendency, or coagulation disorders.
4. Being pregnant or breastfeeding.
5. Combined with fractures in other parts.
6. Fainting during acupuncture or acupuncture intolerance.
7. Mental illness or non-cooperation.
8. Having received acupuncture treatment in the past 2 months.
9. Currently participating in other clinical studies.

### Drop out criteria

1. Having the willingness to withdraw from the study.
2. Suffering from allergy, dizziness, nausea, vomiting, hematoma, and other adverse reactions during the acupuncture process.
3. Having serious complications.
4. Something wrong with the previous diagnosis.
5. Circumstances where the clinician considers it necessary for the patient to withdraw from the experiment.

### Randomization and allocation concealment

Random number tables will be constructed using SPSS 22.0 and run by an independent statistician. All participants will be randomly assigned to the EA group or routine treatment group at a ratio of 1:1. An opaque computer-generated sealed envelope will be used for random allocation. Assignment information is contained in the envelopes, which are numbered sequentially with the serial number on the outside. The envelopes will be opened when the participants finish baseline measurements. The participants will be randomized to the EA group or routine treatment group and will receive interventions based on their group.

### Blinding

Due to the nature of acupuncture, the acupuncturist and participants cannot be blind to allocation. The researchers involved in the evaluation of outcomes will be blinded to the allocation and intervention. Normally, the researchers in charge of evaluation will not be allowed to uncover the blindness.

### Intervention

Subjects in the routine treatment group and the EA group will receive the same medication and rehabilitation treatment. In addition, the EA group will receive additional EA treatment. EA will be conducted in the treatment room of the upper limb inpatient department.

### Routine treatment group

Routine treatment includes medication and rehabilitation. Medication and rehabilitation will be guided by a professional orthopedist and a skilled rehabilitation therapist, respectively.

1. Medication: the first-generation cephalosporin antibiotic cefazolin, ketoprofen tromethamine injection of 30 mg/time, 0.9% sodium chloride injection of 100 ml/time, and vitamin C injection of 2 g/time will be used. These treatments will be administered three times by intravenous drip at 8-h intervals within 24 h after surgery. The Lofenadine sustained-release tablets will be taken orally, one tablet at a time, twice a day, after meals.
2. Rehabilitation treatment: according to the American Orthopedics Association (AAOS) guidelines for post-orthopedic rehabilitation [31], rehabilitation programs following ORIF for DRFs are as follows:
  - Protective period (0–4 weeks): active wrist and forearm range of motion training is performed within 4 days after surgery, and early muscle strength training is performed within 2–4 weeks after surgery. The above training lasts for 10 min each time and is conducted three times a day.
  - Active period (4–8 weeks): strengthening the wrist and forearm movements for 30 min each time, several times a day; moderate resistance and isotonic movement are performed when there is evidence of healing.

### EA group

According to the Therapeutics of Acupuncture and Moxibustion [32], the selected acupoints are located on the affected side, and the main acupoints selected are Yangxi (LI 5), Yangchi (TE 4), Yanggu (SI 5), Hegu(LI 4), and Taiyuan(LU 9). The locations of acupoints are exhibited in Table 1. The location and depth of needling for each point will be in strict accordance with the National Acupoint Standard of the People’s Republic of China in 2021 (GB/T 12346-2021) [33]. The acupuncturist responsible for EA intervention has more than 5 years of practice experience and has undergone training.

**Table 1:** Details of the acupoints used in the EA group

Acupoints	Location
Yangxi (LI 5)	On the radial side of the wrist between the extensor pollicis longus &brevis tendons in a depression formed when the thumb is tilted upward (anatomical snuffbox)
Yangchi (TE4)	On the transverse crease of the dorsum of the wrist between tendons of muscles extensor digitorum and extensor digiti minimi
Yanggu (SI 5)	Near the ulnar end of the transverse wrist crease on the dorsal side of the hand in a depression between the styloid process of the ulna and the triquetral bone
Hegu (LI 4)	On the dorsum of the hand, between the 1st and 2nd metacarpal bones
Taiyuan (LU9)	On transverse crease of the wrist on the lateral side of the radial artery

The patient will be in the supine position. Disposable sterile acupuncture needles with a length of 30 mm and a diameter of 0.25 mm (Beijing Keyuanda Medical Appliance Co., Ltd.) will be used for injection after disinfection. LI 5 and LI 4 will be perpendicularly punctured by 20 mm while TE 4, SI 5, and LU 9 will be vertically pierced by 15 mm. The deqi sensation is induced by thrusting the needle with force and lifting it gently. After Deqi, the EA device (Hwato, Guangzhou Chunliang Medical Appliance Co, Ltd) will be used to connect to points LI 5, TE 4, SI 5, LI 4, and LU 9. The wave will be set as 2/100 Hz. EA will be stopped after 30 min of treatment and all needles will be removed. EA treatment will start on the third day after surgery for fractures. Patients will receive EA treatment on Monday, Wednesday, and Friday for 8 weeks with a 4-weeks follow-up.

### Outcome measures

**Fig. 2:**
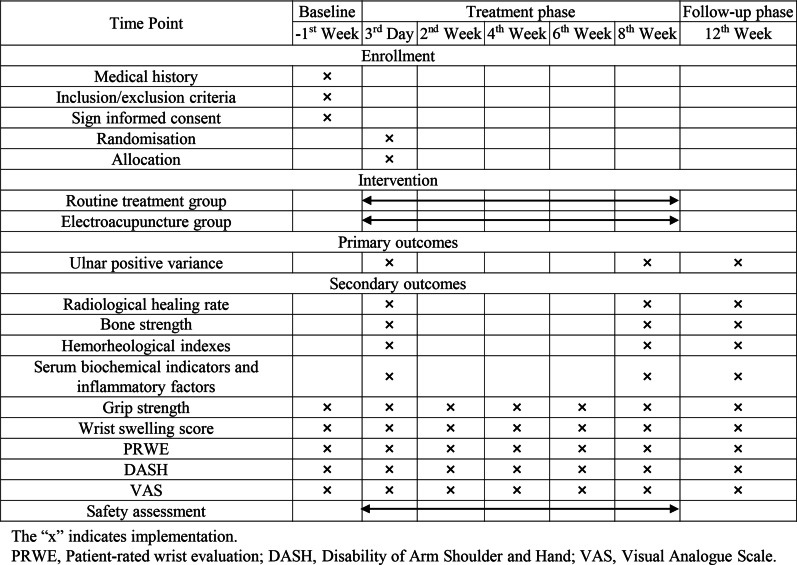
The evaluation time points

The evaluation time points are shown in Fig. 2. There will be a researcher in charge of evaluating the outcomes. Ulnar positive variance, radiological healing rate, bone strength, hemorheological indices, and serum biochemical indicators and inflammatory factors will be measured at 3rd day and 8th week. Other outcomes will be measured at baseline, 3rd day, 2nd, 4th, 6th and 8th weeks after surgery.

### Primary outcome

The primary outcome will be ulnar positive variance. According to the guidelines formulated by American Society of Surgery of the Hand (ASSH, http://www.eradius.com), ulnar positive variance is one of the important radiological parameters for judging the healing of DRFs. Calculating the ulnar positive variance requires making a vertical line of the radial mid-axis through the ulnar lateral edge of the radial articular surface. The minimum distance of the distal ulnar articular surface above this line is measured as the ulnar positive variance.

### Secondary outcome

Secondary outcomes include objective and subjective evaluations. Objective evaluations include radiographic healing rate, bone strength, hemorheological indices, serum biochemical indicators and inflammatory factors, grip strength and wrist swelling score. Subjective evaluations include the patient-rated wrist evaluation (PRWE), disabilities of arm shoulder and hand (DASH), and visual analogue scale (VAS), all of which will be evaluated when evaluating the primary outcome.

1. The radiographic healing rate of DRFs at the 8th week postoperatively is the ratio of the number of radiographic healing cases of DRFs to the number of cases in the group. Radiological healing is defined as trabeculae or bridges of at least one cortical bone (shown on the anterior and posterior plates) and one cortical bone (shown on the lateral plate). All patients are scheduled for radiography at the 8th week. We will calculate the radiographic healing rate based on the results provided by a skilled radiologist.
2. Bone strength will be determined by quantitative ultrasound (QUS) measurements of the bone speed of sound (SOS) along the longitudinal axis of the radial bones in participants. The measured bone strength is expressed as a *z*-value. *Z* < − 2 indicates severe bone strength deficiency. A *z*-value of − 2 to − 1.5 indicates a lack of moderate bone strength. A *z*-value of − 1.5 to − 1 indicates mild bone strength deficiency. *Z* value > − 1 is normal.
3. Hemorheological indices can reflect fracture healing well. Five milliliters of morning blood that have been fasted for more than 12 h will be drawn and the serum will be separated by centrifugation. The total blood viscosity (BV), plasma viscosity (PV), and hematocrit (HCT) of the patients in each group will be measured by an automatic blood rheometer.
4. Serum biochemical indicators and inflammatory factors: (a) Five milliliters of morning blood that has been deprived of food for more than 12 h will be taken and the concentrations of serum calcium, phosphorus, and alkaline phosphatase will be measured by an automatic biochemical analyzer. The ion-selective electrode method is used for the determination of serum calcium (Ca) and the phosphomolybdate method is used for the determination of serum phosphate (P). The alkaline phosphatase concentration will be determined by fluorometry. (b) The levels of high-sensitivity C-reactive protein (hs-CRP), interleukin-1β (IL1β), interleukin-6 (IL-6), and tumor necrosis factor (TNF-α) in the two groups will be determined by an automatic biochemical analyzer.
5. The measurement of grip strength is taken as a direct and non-invasive muscle strength index of the upper limb [34]. The grip strength is measured three times using a calibrated manual ergometer model Jamalplus (Shanghai Yuyan Scientific Instru4ment Co, Ltd). The interval between the two tests should not be too short (more than 15 s). The final result takes its maximum value.
6. The wrist swelling score will be recorded according to the grading standard of soft tissue swelling. The wrist swelling score is divided into four grades: grade 0 (there is no swelling in the wrist), grade 1 (wrist slightly distended, skin lines clear), grade 2 (the wrist shows increased skin tension compared with normal skin, with clear dermatoglyphics, and no tension blister) and grade 3 (the wrist is extremely swollen and the skin has tension blisters) [35].
7. PRWE is an effective test for wrist joint function [36]. The questionnaire consists of three subscales of pain, specific activities, and daily activities, each of which includes 15 items. The overall PRWE includes all 3 subscales and ranges from 0 (no pain or disability) to 100 (maximum pain or disability). PRWE can be used to assess the extent of pain or disability in the wrist of a patient.
8. The DASH score is mainly used to test the degree of disability of the arm, shoulder and hand [37]. The questionnaire includes 30 items to evaluate the physical activity, symptom intensity, and the impact of the injury on social activity. The degree of disability is measured on a scale of 0–100 after scoring, with a higher score indicating greater disability. To make the results true, at least 27 questions must be answered [38]. A negative change from baseline indicates improvement in disability.
9. VAS is the most commonly used scale for measuring pain intensity in pain [39]. The test uses “no pain” and “worst pain possible” as anchor points on a horizontal line (10 cm long). Patients are asked to mark the line that best describes the extent of their pain. On a scale of 0–10, each patient will rate his or her pain (0, no pain; 10, the worst pain imaginable) [40]. A negative change from baseline indicates a decrease in pain.

### Safety assessment

Adverse reactions related to acupuncture may include skin allergy, nerve injury, local infection, broken needles, etc. All adverse events will be documented in detail in the case report forms (CRFs) and reported to the Medical Ethics Committee, including the time, frequency, and cause of the adverse events. When a serious adverse event occurs, and a causal relationship with treatment cannot be ruled out, the primary researcher will determine whether to suspend treatment.

### Provisions for post-trial care

We are willing to provide the routine treatment group with free EA treatment for 1 month after the trial. Besides, every participant will also receive a document during their treatment. This document relates to adjuvant care for subjects experiencing adverse reactions during the intervention. If necessary, we will provide corresponding supplementary care for participants.

### Data collection and management

The researcher involved in the evaluation will receive strict training to ensure the accuracy of the measurement of the outcomes. Two research assistants are responsible for inputting all data into the CRFs after the measurement is completed. If the participants give up participating in the trial halfway, we will record the reasons and use the latest data for analysis. The informed consent signed by the participants will be stored in the special file management folder of the Department of Upper Limbs, Shenzhen Pingle Orthopedic Hospital. The measured data will be updated once a week. Data are stored on the mobile hard disk and backed up in Baidu Cloud. Data will not be made public during the course of the study. All data are archived for 10 years after the end of the study.

### Data monitoring

There will be a data monitoring committee to regularly monitor the trial data to protect the interests of the subjects. The research assistants need to upload the entered data to the data monitoring committee, including information of participants, informed consent, and data of primary and secondary outcomes. The data monitoring committee will decide whether to modify or terminate the trial according to the uploaded data. The final review results will be sent to the trial researchers. The chair is Mr. Zhang JP, with Dr. Liu HC, and Dr. Huang ST.

### Sample size calculation and statistical analysis

Based on previous experiments [41], the positive variance of the ulna decreased by 0.6 mm, and the standard deviation was 1.81 mm after the treatment of DRFs. Therefore, we assume that the difference in the positive variance of the ulna between the EA group and the routine treatment group at the 8th week after ORIF is 0.6 mm, and the standard deviation is 1.81 mm. Calculations will be performed using SAS (SAS 9.4 Institute, SAS, Cary, NC) based on a two-tailed alpha error of 5% and with a statistical power of 80%. The calculated sample size is based on the following equation: $N = \frac{{2 \times \frac{Z\alpha }{2} + Z_{\beta }^{2} \times \sigma^{2} }}{{\delta^{2} }} = \frac{{2 \times \left( {1.96 + 0.25} \right)^{2} \times 1.81^{2} }}{{0.6^{2} }}$. The result is a minimum of 89 participants per group. Considering the 20% drop-out rate, we plan to enroll a total of 222 participants, 111 participants per group.

All data will be counted and analyzed by an independent researcher who does not participate in the trial. Statistical analysis will be performed using SAS (SAS 9.4 Institute, Cary, NC) based on the principle of intentional treatment. Missing data will be interpolated using multiple interpolation. Statistical significance is defined as a two-sided *p*-value < 0.05. Descriptive characteristics of baseline statistics will be reported as mean standard deviation or median (interquartile range) [M(IQR)]. Since all results will be evaluated at multiple time points postoperatively, statistical analysis will be performed based on the results from the same week in both the EA group and the routine treatment group. Among them, the radiographic healing rate will be tested by *χ*2 test. Other quantitative data will first be tested for normality using Kruskal–Wallis. If the distribution of data is normal, it will be tested by repeated measurement analysis of variance (rm-ANOVA), including between-group and within-group effects. If the data distribution is skewed, the linear mixed model is adopted.

## Discussion

Surgical treatment is one of the common treatments for DRFs [42]. Rehabilitation and medication are routine treatments after surgery. However, these treatments are controversial [43]. Acupuncture, as a therapy with the characteristics of traditional Chinese medicine (TCM), has a wide range of clinical applications due to its good efficacy and low side effects. Some clinical studies have reported the positive effect of acupuncture on patients with DRFs [20, 44]. However, studies related to acupuncture promoting fracture healing had a limited sample size and included low-quality randomized controlled trials reported by a recent meta-analysis [4]. Therefore, the evidence of acupuncture promoting postoperative healing of DRFs is still uncertain, and more high-quality, large-sample, and well-designed randomized controlled trials should be conducted to provide a reliable basis for further verification. In this protocol, we design a scientific and rigorous trial to explore the effects of EA on the postoperative healing of DRFs. The healing time of DRFs is usually 12 weeks [45], so we design a trial with an 8-weeks treatment and a 4 weeks follow-up. Based on “Therapeutics of Acupuncture and Moxibustion” [32] and previous studies [20, 46, 47], Yangxi(LI 5), Yangchi (TE 4), Yanggu (SI 5), Hegu (LI 4), and Taiyuan (LU 9) are the most frequently selected acupoints for the treatment of wrist sprain. Referring to the previous research and clinical practice [15, 17, 21], we carry out EA treatment on the third day after surgery. In order to promote blood circulation and eliminate inflammatory edema, we choose the density wave with a frequency of 2/100 HZ. Previous guidelines developed by ASSH and several studies have shown that ulnar variability is an accurate radiological predictor of functional outcome [48, 49]. We will calculate the changes in ulnar positive variance before and after treatment in both groups of patients. This difference may provide more sufficient evidence for determining whether EA is effective in treating DRFs. PRWE and DASH have been demonstrated to be effective and reliable measures of function and disability in patients with displaced DRFs [50]. Patients often suffer from poor local blood supply and slow healing after fracture surgery. Besides, pain and swelling have always plagued patients. Therefore, we design this trial to understand the possible functional characteristics of EA and in which aspect it helps fracture healing, to provide better evidence reference for clinical practice. There are some limitations in our study. Due to single-institution data, the results of our study may be biased and unrepresentative. In addition, our experimental design includes multiple outcomes, which requires good cooperation of participants. These limitations should be improved in our future studies. In conclusion, to achieve our clinical goals, we will strive to standardize each step of the study, including acupoint selection, acupuncture manipulation, result evaluation, and so on. We anticipate that this trial will provide an evidence-based treatment solution for patients after surgery for DRFs.

## Data Availability

Not applicable.

## Abbreviations

DRF: Distal radius fracture
EA: Electroacupuncture
PRWE: Patient-rated wrist evaluation
DASH: Disabilities of arm shoulder and hand
VAS: Visual analogue scale
AAOS: American Orthopedics Association
QUS: Quantitative ultrasound
SOS: Speed of sound
BV: Blood viscosity
PV: Plasma viscosity
HCT: Hematocrit
Ca: Calcium
P: Phosphate
hs-CRP: High-sensitivity C-reactive protein
IL-1β: Interleukin-1β
IL-6: Interleukin-6
TNF-α: Tumor necrosis factor
CRFs: Case report forms
M(IQR): Median (interquartile range)
rm-ANOVA: Repeated measurement analysis of variance
TCM: Traditional Chinese medicine
ASSH: American Society of Surgery of the Hand

## Declarations

### Tiral status

The first patient will be recruited on 1 December 2022. This study will complete the recruitment of all patients in March 2025. The protocol (version 1) has been registered at China Clinical Trials Registry on August 21, 2022 (No. ChiCTR2200062857).
